# A Phase I vaccine trial using dendritic cells pulsed with autologous oxidized lysate for recurrent ovarian cancer

**DOI:** 10.1186/1479-5876-11-149

**Published:** 2013-06-18

**Authors:** Lana E Kandalaft, Cheryl L Chiang, Janos Tanyi, Greg Motz, Klara Balint, Rosemarie Mick, George Coukos

**Affiliations:** 1 Ovarian Cancer Research Center, University of Pennsylvania, Philadelphia, PA, USA; 2 Department of Biostatistics and Epidemiology, Perelman School of Medicine, University of Pennsylvania, Philadelphia, PA, USA

**Keywords:** Recurrent ovarian cancer, Immunotherapy, Tumor vaccine, HOCl, Bevacizumab, Cyclophosphamide

## Abstract

**Purpose:**

Ovarian cancer, like most solid tumors, is in dire need of effective therapies. The significance of this trial lies in its promise to spearhead the development of combination immunotherapy and to introduce novel approaches to therapeutic immunomodulation, which could enable otherwise ineffective vaccines to achieve clinical efficacy.

**Rationale:**

Tumor-infiltrating T cells have been associated with improved outcome in ovarian cancer, suggesting that activation of antitumor immunity will improve survival. However, molecularly defined vaccines have been generally disappointing. Cancer vaccines elicit a modest frequency of low-to-moderate avidity tumor-specific T-cells, but powerful tumor barriers dampen the engraftment, expansion and function of these effector T-cells in the tumor, thus preventing them from reaching their full therapeutic potential. Our work has identified two important barriers in the tumor microenvironment: the blood-tumor barrier, which prevents homing of effector T cells, and T regulatory cells, which inactivate effector T cells. We hypothesize that cancer vaccine therapy will benefit from combinations that attenuate these two barrier mechanisms.

**Design:**

We propose a three-cohort sequential study to investigate a combinatorial approach of a new dendritic cell (DC) vaccine pulsed with autologous whole tumor oxidized lysate, in combination with antiangiogenesis therapy (bevacizumab) and metronomic cyclophosphamide, which impacts Treg cells.

**Innovation:**

This study uses a novel autologous tumor vaccine developed with 4-day DCs pulsed with oxidized lysate to elicit antitumor response. Furthermore, the combination of bevacizumab with a whole tumor antigen vaccine has not been tested in the clinic. Finally the combination of bevacizumab and metronomic cyclophosphamide in immunotherapy is novel.

## Background and rationale

Ovarian cancer cells are antigenic and express a multitude of known tumor-associated antigens (TAAs) including Her-2/neu [1-3], p53 [4], NY-ESO-1 [5,6], cdr2 [7], hTERT [8,9], mesothelin [10], survivin [11,12] SP-17, WT1 [13-19] etc. Clinical data now clearly indicate that the immune system affects the outcome of patients with epithelial ovarian cancer (EOC). We and others have shown that the presence of intraepithelial tumor-infiltrating lymphocytes correlates with improved progression-free and overall survival [20-28]. TILs isolated from ovarian cancers are oligoclonal [29,30], recognize autologous tumor and known TAAs in vitro [14,31-34], and exhibit tumor-specific cytolytic activity ex vivo [35,36]. Tumor-specific T-cell precursors can also be detected in the blood of patients with advanced ovarian carcinoma [37]. These observations suggest that activation of antitumor immunity could be feasible and could produce clinical results. To date, immunotherapy investigations have yielded limited but encouraging results in EOC. For example, weekly intraperitoneal IL-2 infusion produced a ~17% complete pathologic response rate in ovarian cancer [38,39], while anecdotal objective responses have been reported with CTLA-4 antibody [40,41]; adoptive transfer of TILs [23,24]; or vaccines using NY-ESO-1 peptide [42], virus-modified autologous tumor cells [43] or DCs pulsed with whole autologous tumor lysate [44].

## Whole tumor cancer vaccines

Therapeutic cancer vaccines have the potential to break immune tolerance and induce long-term immune response against cancer cells. However, molecularly defined vaccines directed towards known TAAs have either failed to produce clinical responses or have yielded transient responses in ovarian cancer patients to date, as none of the above antigens, except for NY-ESO-1, have been proven to be *bona fide* rejection antigens in the clinic [42,45,46]. A reasonable alternative may be whole tumor vaccines [47-49]. The advantages of these were reviewed recently [50]. Tumor cells express a whole array of antigens, most of which remain uncharacterized in EOC. Vaccination with whole tumor antigen potentially draws on this rich source of antigens, comprising epitopes for both CD8^+^ cytotoxic T-cells (CTLs) as well as CD4^+^ T helper (Th) cells, a possibly necessary condition to ensure tumor homing of low affinity CD8^+^ cells [51-53]. Whole tumor vaccines could also greatly diminish the chance of tumor escape compared to single epitope vaccines. Finally, recent deep sequencing results from over 300 advanced EOC specimens show that ovarian tumors carry an average of 61 somatic non-synonymous mutations, most of which were private [54]. Some of these mutations could potentially give rise to neo-antigens that could stimulate effective and long-lasting anti-tumor responses. Interestingly, a meta-analysis of 173 published peer-reviewed immunotherapy trials found that 8.1% of patients vaccinated with whole tumor antigen (n=1,733) experienced objective clinical responses, compared with 3.6% of patients vaccinated with defined tumor antigens (n= 1,711; *P* < 0.0001) [55]. Although, studies have shown that whole tumor lysates can be poorly immunogenic and can suppress DC differentiation and maturation [56-58], some approaches to lysate preparation can increase immunogenicity of whole tumor lysates [59-61]. In this study, we exploit oxidation during the preparation of tumor lysate, which appears to promote immunogenicity [62].

## A novel approach to tumor cell lysate preparation

A widely used and straightforward method of whole tumor cell preparation already used in clinical trials is necrotic whole tumor cell lysate. The efficacy of the necrotic cell lysate can be further enhanced by oxidative modification using hypochlorous acid (HOCl) treatment [59]. It has been demonstrated that proteins oxidized by HOCl are more readily taken up and processed by antigen presenting cells (APCs) and lead to enhanced priming of autologous tumor-specific CD4^+^ and CD8^+^ T-cell responses in vitro [63-66]. The use of HOCl to potentiate the immunogenicity of whole ovarian tumor cells has been evaluated using SKOV3 ovarian cancer cells [67,68]. The improvement in antigen immunogenicity is explained by three possible mechanisms. First, HOCl can quantitatively deaminate serine and convert its side chain into an aldehyde, leading to significant improvement in immunogenicity [69-71]. Second, oxidation of protein antigens might allow protein unfolding and exposure of cryptic immunogenic peptides to specific T-cells [72]. Third, scavenger receptors such as the lectin-like oxidized low-density lipoprotein receptor-1 (LOX-1) might be involved in the uptake of HOCl-oxidized tumor cells [73-75], leading to DC activation and efficient presentation of MHC-I as well as MHC-II restricted peptides [59,76]. In preclinical evaluation, this tumor lysate preparation proved to be more immunogenic than the standard UV treated whole tumor lysate [50]. This will be the first study utilizing DCs pulsed with oxidized whole tumor lysate.

## A new dendritic cell vaccine platform

DCs loaded with whole tumor lysate have been investigated in several clinical trials for their ability to induce anti-tumor T-cell responses [44,77-80]. Beneficial anti-tumor responses have been observed in some patients, illustrating the potential of this approach. DCs can be classified into different subsets, depending on their lineage and receptor expression pattern. Their distinct biology can be exploited for different therapeutic strategies. The most widely used DCs in clinical trials to date are myeloid DCs differentiated from peripheral blood monocytes. In most trials, “classic” DCs are fully differentiated over seven days in the presence of recombinant granulocyte-macrophage colony stimulating factor (GM-CSF) and interleukin 4 (IL-4) [81-83]. These DCs exhibit high phagocytic and antigen-processing capability. Upon maturation with an appropriate stimulus, Day-7 DCs up regulate costimulatory surface molecules such as CD80, CD86, CD40, and lymph node-homing receptors such as CCR7, and can efficiently prime naïve T-cells [84-86].

We developed a faster, four-day protocol for DC preparation, using GM-CSF, IL-4 and serum-free AIM-V media that is suitable for clinical use. We showed that Day-4 DCs generated with this protocol are similar to “classic” Day-7 DCs, in terms of phenotype and phagocytic capability, and have a higher capacity than Day-7 DCs to produce IL-12p70 following LPS and IFN-γ stimulation. In addition, these Day-4 DCs were highly immunogenic, and efficiently primed ovarian tumor-specific T-cells in vitro in peripheral blood lymphocytes from healthy volunteers and ovarian cancer patients [87].

## Enhancement of immune therapy by antiangiogenic therapy

It has been shown that vascular endothelial growth factor (VEGF) suppresses tumor antigen presentation through blockade of myeloid DC differentiation and maturation, leading to tumor immune tolerance [88-95], a process primarily mediated by VEGF receptor-1 (VEGFR-1) [96]. VEGF also up regulates programmed death ligand 1 (PD-L1 or B7-H1) in myeloid DCs, which is associated with T-cell suppression and exhaustion [97]. VEGF blockade restores DC function and enhances immunotherapy [12,98-101]. Anti-VEGF strategies reduce the number of CD4^+^CD25^+^ T regulatory (Treg) cells when administered in combination with a GM-CSF secreting tumor vaccine, resulting in increased CTL induction and improved vaccine efficacy [102,103]. Similar effects were observed in human subjects treated with VEGF-neutralizing antibody therapy [98]. A single-arm clinical trial of vaccine and bevacizumab for prostate cancer has shown that the combination is associated with a high rate of immune response induction [104].

Work by us and others have demonstrated that angiogenesis mechanisms also impair the effector arm of antitumor immunity by blocking homing of effector T-cells into tumors. This is in part mediated through the endothelin B receptor (ET_B_R), which is up regulated in tumor endothelium and deregulates endothelial ICAM-1 expression and T-cell adhesion. This blood-tumor endothelial barrier can be disrupted by ET_B_R antagonists, resulting in dramatic increase of T-cell homing in tumors and significant efficacy of otherwise ineffective vaccine therapy in the mouse, thus calling for human experimentation [105,106]. Importantly, ET_B_R is upregulated by VEGF. In vitro, ET-1 signaling through ET_A_R in cancer cell lines leads to de novo production of VEGF, a process regulated by HIF-1-alpha [107] utilizing initial PGE_2_ production [108] as an intermediary before secondary VEGF production [107,109-112]. We confirmed that VEGF blockade enhances T-cell homing to tumors in ID8-VEGF tumors, a murine syngeneic model of ovarian cancer overexpressing VEGF [113]. Following vaccination with UV-irradiated tumor cells, in spite of a tangible frequency of antitumor T-cells in the spleen, no CD8^+^ TILs were detected in ID8-VEGF tumors. In agreement with others [114], treatment with the VEGFR-2 tyrosine kinase inhibitor SU5416 [115] produced a dramatic influx of CD8^+^ TILs in ID8-VEGF tumors, while DMSO vehicle had no effect on TILs (unpublished data). Normalization of tumor vasculature through disruption of the VEGF/VEGFR-2 axis was also shown to increase extravasation of adoptively transferred T-cells into the tumor and improve adoptive cell transfer immunotherapy in a murine cancer model [116]. Combining VEGFR-2 antibody DC101 with Her-2-specific vaccination in a mouse model of Her-2/*neu*-induced breast cancer, it was demonstrated that this combination treatment accelerated tumor regression augmenting the lytic activity of CD8^+^ cytotoxic T-cells [114]. Thus, VEGF blockade not only blocks tumor angiogenesis, but may also increase the efficacy of tumor vaccines by enhancing DC function and by increasing T-cell homing to tumors.

## Using metronomic chemotherapy to enhance immune response

Although, the traditional view has been that chemotherapy may neutralize antitumor immune response generated through vaccine therapy, emergent data indicate that chemotherapy can be combined safely with immunotherapy with possibly additive or synergistic effects that are dose and schedule dependent. The most extensively investigated drug to enhance vaccine potency is cyclophosphamide, a drug previously used in ovarian cancer as standard of care in combination with cisplatin. One of the first observation was made by Berd and colleagues who used a regimen of low-dose cyclophosphamide (300 mg/m^2^ i.v.), given three days prior to vaccination with autologous melanoma cells admixed with Bacillus Calmette-Guerin (BCG), to treat patients with melanoma [117]. They reported that cyclophosphamide plus vaccine treatment resulted in a progressive depletion of circulating CD4^+^ suppressor T-cells.

In mouse models, North presented evidence that intravenous cyclophosphamide enhanced tumor immunotherapy by elimination of CD8^+^ tumor suppressor cells [118], and that the effect was independent of direct tumor cytotoxic effects [119]. Machiels and colleagues evaluated the combination of cyclophosphamide with (GM-CSF)–secreting whole-cell vaccines in the HER-2/*neu* mouse model of mammary cancers [120]. They found that when cyclophosphamide was given at a dose range between 50 and 150 mg/kg 1 day prior to vaccine, the combination controlled tumors more effectively than either agent alone. Subsequent work showed that cyclophosphamide depletes Treg cells [121,122] and it impairs their function for nearly 10 days post treatment.

Cyclophosphamide has been used also in humans to augment cancer immunotherapy [123,124]. In patients with advanced colorectal carcinoma and melanoma, cyclophosphamide was shown to increase the response to an adjuvant KLH vaccine. Jaffee and colleagues have examined carefully the dose-dependent immunomodulatory effects of cyclophosphamide with respect to targeting T regulatory (Treg) cells [125]. Emens et al. conducted a Phase I trial that evaluated allogeneic, Her-2-positive GM-CSF–secreting breast tumor vaccine alone or in sequence with low doses of cyclophosphamide and doxorubicin in metastatic breast cancer patients (n=28), and they found that the dose of 200 mg/m^2^ of intravenous cyclophosphamide augmented Her-2-specific humoral immunity. However, the immunomodulatory effect of intravenous cyclophosphamide was lost if given at doses above 200 mg/m^2^[125]. Single-agent intravenous cyclophosphamide doses of 150, 250, and 350 mg/m^2^ were also evaluated in patients with hepatocellular carcinoma by Greten et al. who reported that the lower doses (150 and 250 mg/m^2^) induced a decrease the number and the relative frequency of circulating regulatory T-cells, and that the dose of 250 mg/m^2^ was able to impair the suppressor function of regulatory T-cells. It was also shown in a phase I trial of pancreatic cancer patients that inhibition of regulatory T-cells (when using 250 mg/m^2^ intravenous cyclophosphamide) resulted in recruitment of high-avidity effector T-cells to tumors, leading to prolonged progression-free survival and overall survival [126].

It has been hypothesized that combining VEGF blockade with low-dose (metronomic) chemotherapy may have positive antiangiogenic or antitumor effects [127]. In a multi-institutional phase II study (NCI-5789), 29 subjects were treated with bevacizumab 10 mg/kg every 14 days and low-dose oral cyclophosphamide 50 mg daily [128]. The response rate was 28% and 6-month progression free survival rate of 57%. A second phase II prospective study investigated the efficacy and safety of intravenous bevacizumab 10 mg/kg every other week plus oral cyclophosphamide 50 mg daily in fifteen heavily pretreated patients with recurrent ovarian cancer [129]. There was significant activity, with a response rate of 53%. Despite being heavily pretreated, no gastrointestinal perforations were noted. In short, combination of bevacizumab and cyclophosphamide is a promising dual antiangiogenic/tumoristatic and immunomodulatory therapy that is available at the present time. The rationale for combining this therapy with vaccine is to prime the tumor microenvironment and create a favorable milieu to achieve greater efficacy and immune response.

## Trial design

**Figure 1:**
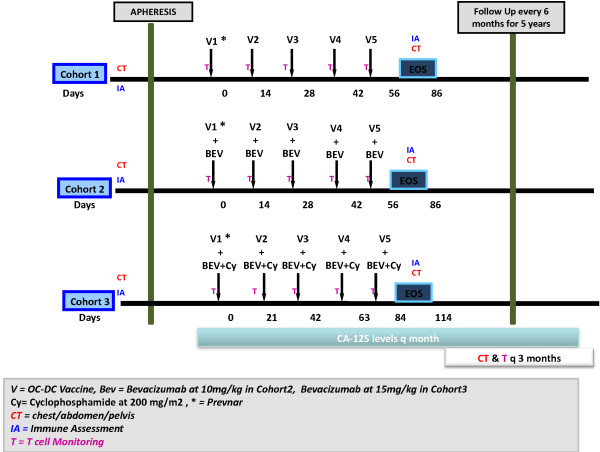
Clinical trial design.

We propose a phase I, three-cohort, single-center study to establish the safety and proof of concept of Oxidized tumor Cell pulsed DC (OCDC) vaccine administered intranodally, alone (Cohort 1, n=5 subjects) or in combination with either intravenous bevacizumab (Cohort 2, n=10 subjects) or intravenous bevacizumab and intravenous cyclophosphamide (Cohort 3, n=10 subjects) in subjects with recurrent ovarian, fallopian tube or primary peritoneal cancer (Figure 1). Inclusion criteria require patients 18 years or older, diagnosed with advanced stage disease, with ECOG performance status ≤1, and >6 months expected survival.

**Figure 2:**
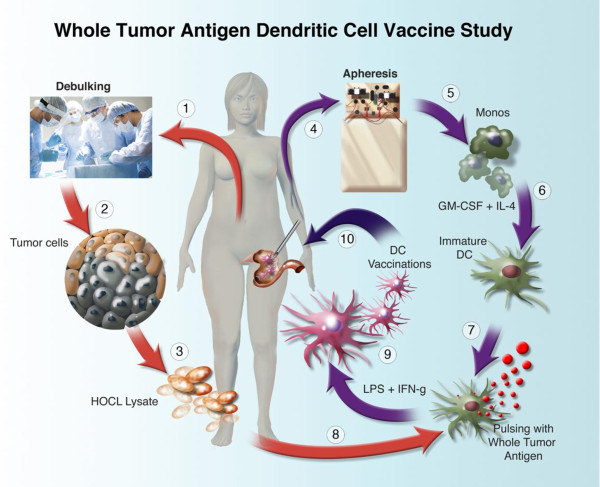
Clinical trial schema.

The components of the vaccine in this study include agents, for which safety has been previously demonstrated to be acceptable. During this current study we intend to manufacture a modified whole tumor vaccine using oxidized whole tumor cell lysate derived from autologous tumor harvested at secondary debulking surgery, pulsed onto autologous dendritic cells (Figure 2). The choice of dendritic cell maturation and the choice of tumor cell preparation are based on previously published data [67,130].

## Regimen

Eligible patients will undergo a 10–15 liter apheresis around day −30 to −15 to harvest peripheral blood mononuclear cells (PBMC) for DC manufacturing. Patients will receive OCDC tumor vaccine in combination with other agents in a design that escalates combinatorial complexity. OCDC is prepared at the Cell and Vaccine Production Facility of the University of Pennsylvania. It will be released in sterile syringes containing ~2.5-5 × 10^6^ DC in 0.55 mL sterile saline. Subjects will receive a total dose of approximately 5–10 × 10^6^ DCs administered through two or more intranodal injections into different normal groin nodes; 0.55 cc containing ~2.5-5 × 10^6^ DCs will be injected per node with a 22 Gauge needle.

Subjects in Cohort 1 will receive five doses of 5–10 × 10^6^ dendritic cells (OCDC vaccine) intranodally, while subjects in Cohort 2 will receive the same five doses of vaccine in combination with intravenous bevacizumab (10 mg/kg) given every two weeks on days 0, 14, 28, 42 and 56. Subjects in Cohort 3 will receive the same five doses of vaccine on days 0, 14, 28, 42 and 56, while bevacizumab (10 mg/kg) plus cyclophosphamide (200 mg/m^2^) will be given the day before each vaccination.

Subjects will be offered to undergo apheresis within two weeks after vaccine #4 or one to three weeks after vaccine #5 to collect vaccine primed PBL for use in a follow-on study of adoptive T-cell therapy. Each treatment cohort will be evaluated separately, to detect any side effects that may be due to the vaccine in combination with other biological agents. Patients in Cohort 2 will be taken off bevacizumab, if they experience severe adverse events (SAEs) at least likely related to bevacizumab, but will retain the option of continuing OCDC vaccination on the study. Subjects in Cohort 3 will be taken off bevacizumab/cyclophosphamide, if they experience severe adverse events (SAEs) at least likely related to bevacizumab or cyclophosphamide, but will retain the option of continuing OCDC vaccination on the study. Subjects will be taken off the study completely if they experience SAEs, which are possibly, probably or definitely related to OCDC. Termination of enrollment for each cohort will be triggered with ≥2 DLTs (i.e., DLT is any Grade 3 or higher allergic, autoimmune or injection site reaction or any Grade 4 hematologic or non-hematologic toxicity expect fever) in the first 5 subjects and ≥3 DLTs at any time. The rules for early stopping for toxicity do not depend on the availability of immune response data; any subject that gets at least one vaccination injection is included. For this study, 25 evaluable subjects will be treated, and we estimate that up to 30 subjects may need to be enrolled, assuming a 20% failure rate for generating the dendritic cell vaccine.

Subjects will be followed daily for the first 5 days (starting at Day 0), and then biweekly until week 8 (End of Study). At screening and 30 days following the fifth vaccine dose (day 86), subjects will undergo immune assessment. Immune monitoring will be performed on blood samples from all participating patients to assess the vaccine induced antitumor immune response and the composition of circulating T-cell subpopulations. This study obtained approval from national health agencies and from the Institutional Review Board of the University of Pennsylvania and is performed in accordance with the Helsinki Declaration, the International Conference of Harmonization Good Clinical Practice guidelines, and local regulatory requirements. Written informed consent will be obtained from each patient.

## Objectives

The primary objective of the study is to determine the feasibility and safety of administering OCDC intranodally alone, in combination with intravenous bevacizumab, or in combination with intravenous bevacizumab plus cyclophosphamide in subjects with recurrent ovarian, fallopian tube or primary peritoneal cancer. The secondary objectives of the study are to obtain pilot data on immunogenicity on OCDC administered intranodally in subjects, to assess the effect of the proposed treatments on peripheral blood regulatory T-cells and on the tumor microenvironment, as well as to evaluate clinical responses up to 114 days.

## Statistical methods

Toxicity will be graded by NCI Common Toxicity Criteria (NCI-CTC) Version 4.0 and will be tabled by treatment cohort. Immune response will be evaluated by descriptive statistics, scatter plots of pre- and post-vaccine values of the various immune parameters, and relevant fold changes by treatment cohort. Exploratory longitudinal analyses (repeated measures ANOVA or linear mixed effects models) will be used to examine time trends (e.g., decrease in Treg cells), test for differences between baseline and post-vaccine time points within cohorts and discern differences among cohorts. Clinical responses scored by RECIST criteria will be tabulated by treatment cohort.

## Innovation

The proposed first-in- human study is innovative in many ways. First, it translates novel concepts of combinatorial immunomodulation of the tumor microenvironment from the laboratory to the clinic. The notion of blocking VEGF in combination with the whole tumor cancer vaccine is novel and has not been tested in the clinic. Preclinical data in solid tumor mouse models show that blocking VEGF enhances de novo T-cell infiltration into the tumor [114] and, when combined with cancer vaccines or adoptive T-cell therapy, VEGF blockade significantly improves their biological and clinical efficacy [104,116,131]. Furthermore, although countering Treg cells in combination with vaccine is not novel, the suppression of Treg cells (by low-dose cyclophosphamide) followed by VEGF blockade is novel in the context of immunotherapy. This drug combination could address the possibility that VEGF blockade could trigger tumor hypoxia, which in turn could induce Treg recruitment and immune tolerance via CCL28 chemokine, as we recently reported [132]. Finally, the vaccine platform used in this study is innovative in its own right. Following careful optimization in the lab [62,87], we are proposing an autologous vaccine with DCs developed from elutriated monocytes cultured for only four days with GM-CSF and IL-4 and pulsed with lysate of HOCl-oxidized tumor cells, which have not been used in the clinic before. DCs will be matured with LPS/IFN-γ and injected intranodally. Collectively, this approach is novel for ovarian cancer, a disease in dire need of new therapies.

## Discussion

We propose a new combinatorial therapy approach to mobilize antitumor immunity against ovarian cancer. The strong association between the presence of intraepithelial T-cells along with other biomarkers of immune activation in tumors and improved clinical outcome suggests that mobilization of antitumor immunity should yield clinical benefit in many patients with EOC, a notion preliminarily supported by many published pilot studies. Our group and other groups have however, revealed the existence of numerous and overlapping mechanisms of immune dysfunction in ovarian cancer, which will have to be abated in order to effectively mobilize antitumor immunity. The combinatorial approach proposed herein is a first attempt to utilize readily available therapeutic tools with known clinical and biological behavior to address some of the tumor barriers.

Given the paucity of reliable tumor rejection antigens in EOC (with the exception of NY-ESO-1, which is relatively rare), we propose to use a whole tumor lysate vaccine. Given the easy accessibility of primary but also recurrent tumors in the peritoneal cavity, and the general acceptance of primary or secondary surgical cytoreductive surgery as the standard of care for these patients, autologous tumor lysate is a feasible approach in this population. Autologous tumor lysates provide a convenient and personalized source of multiple tumor antigens, possibly encompassing all the relevant class I and II epitopes against which antitumor immune response can be mounted, including private and mutated epitopes. Incorporation of class II epitopes could be especially important, since the coexistence of tumor-reactive CD4^+^ cells can enhance tumor engraftment and persistence of low affinity anti-tumor CD8^+^ cells [53]. The choice of tumor cell preparation is based on previous data demonstrating that oxidative necrosis enhances the immunogenicity of whole tumor cell lysates and is superior to other conventional tumor lysates [133,134].

There is no strictly defined standard duration of culture to generate human PBMC-derived DCs. To date, most clinical studies have used a 7-day culture with GM-CSF and IL-4. However, data from Czerniecki and colleagues have shown that fully functional antigen-presenting cells (APC) can be rapidly developed from CD14^+^ PBMC cells in as little as 40 hours [135,136]. Although, these “rapid DC” are efficient in presenting class I and II peptides, data from our laboratory revealed that at least four days of differentiation with GM-CSF and IL-4 were required for elutriated peripheral blood monocytes to acquire the phenotype and functional properties of cross-presenting APCs capable of processing lysate antigen [87]. These 4-day DCs pulsed with lysate underwent proper maturation into DC1 cells when exposed to bacterial lipopolysaccharide (LPS) combined with IFN-γ, producing high levels of IL-12, but required a concomitant – not sequential – exposure to the two maturing agents [62]. We selected these cells as the proposed vaccine platform, which will be administered intranodally, since intranodal administration of DCs allows administration of a defined quantity of DCs directly to the site of T-cell sensitization. This approach also allows the peak IL-12 secretion to be synchronized with their proximity to T-cells, where IL-12 can exert its full effects during antigen presentation [137]. IL-12 is paramount as dendritic cells that are able to produce high levels of IL-12 can induce long-lived type 1 T-cell responses against tumor-associated antigens more efficiently than standard mature DCs. The benefit of high IL-12 producing DCs was highlighted in several recent papers [138-140] demonstrating the importance of IL-12 production with regard to the induction of tumor-specific CTLs in vitro and its ability to predict prolongation of progression-free survival of patients with advanced cancer [141]. Okada et al. showed in a Phase I/II cancer vaccine trial of malignant glioma patients that IL-12 production levels by αDC1 positively correlated with time to progression.

In a murine tumor model, DC pulsed with tumor lysate and injected intranodally resulted in greater sensitization of T-cells and improved anti-tumor responses [142]. In a randomized, Phase I, dose-escalation trial Lambert et al. compared different administration routes (intravenous, intranodal, intradermal) in metastatic melanoma receiving four autologous peptide-pulsed DC vaccinations. The results showed that intranodal administration led to superior T-cell sensitization as measured by de novo target-cell recognition and DTH priming, indicating that intranodal injection may be the preferred route of administration for mature DC vaccines [143].

Adoptive immunotherapy has frequently resulted in tumor rejection in the human, suggesting that a critical number of high-avidity tumor-reactive T-cells are probably required to effectively overcome barriers in the tumor microenvironment. On the contrary, cancer vaccines have most commonly failed to induce overwhelming tumor responses in patients. Preclinical models show that if one can defeat immune barriers in the tumor microenvironment, one can enable low-avidity/low-frequency antitumor immune responses induced by vaccines to become clinically effective. Work from our lab and from other labs shows that among tumor microenvironment barriers preventing the engraftment, expansion and function of antitumor effector T-cells, two can be readily targeted in ovarian cancer: a) angiogenesis driven by VEGF, and b) Treg cells.

Vascular endothelial growth factor is highly expressed and plays an important role in tumor progression of ovarian carcinoma. Positive immunostaining for VEGF was observed in 97% (68 out of 70) of ovarian carcinomas [144] and high VEGF levels correlated with advanced disease stage and poorer survival [144,145]. VEGF became a fundamental target in anti-angiogenic therapy leading to the development of humanized recombinant monocloncal antibody bevacizumab, which was evaluated for ovarian cancer treatment in clinical trials only recently. In the OCEANS study, a phase III randomized study, platinum-sensitive recurrent ovarian and fallopian tube cancer subjects (n=484) were randomized to the combination of gemcitabine and carboplatin either with or without bevacizumab for 6 to 10 cycles. The study showed that the combination therapy including administration of bevacizumab until disease progression resulted in a statistically significant improvement in progression-free survival (hazard ratio: 0.484, p<0.001) [146]. Bevacizumab was also evaluated as frontline therapy for patients in the randomized Phase III ICON7 trial, where stage IIIc or IV patients (n=1528) were randomized to carboplatin and paclitaxel with or without bevacizumab given concurrently every 3 weeks for 5 or 6 cycles and continued for 12 additional cycles or until progression of disease. The ICON7 study has demonstrated that bevacizumab improved the progression-free survival in women with high-risk ovarian cancer (hazard ratio: 0.84, p=0.004). The benefits with respect to both progression-free and overall survival were greater among those at high risk for disease progression [147].

**Figure 3:**
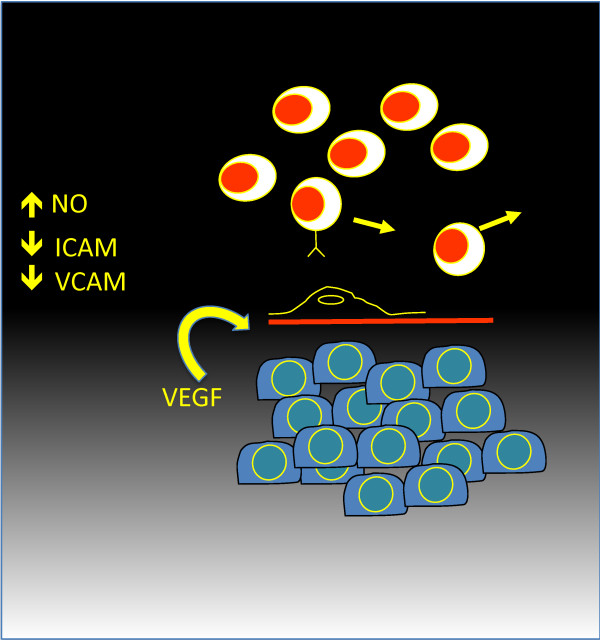
VEGF blockade and the endothelial blood-tumor barrier.

Therefore, VEGF blockade can provide a) important antiangiogenic therapeutic effects, b) attenuate the endothelial blood-tumor barrier (Figure 3) and c) improve DC maturation. Tumor vascular endothelium is a physicial barrier through which T-cells home to the tumor and can present a significant challenge to the success of immunothery. Tumor endothelial cells regulate leukocyte trafficking via adhesion molecules and chemokines [148]. The adhesive properties of tumor endothelium can be deregulated by signaling through the endothelin-B receptor, resulting in the inability of T-cells to adhere and home effectively to tumors [105]. It is possible that some of these effects are maintained by VEGF, since VEGF blockade can enhance T-cell adhesion to endothelium and T-cell homing to tumors [149,150]. Endothelial cells can also express surface inhibitory or death ligands mediators including PDL-1 and PDL-2 [151,152], Fas ligand (FasL; also known as CD95L) [153], TNF-related apoptosis-inducing ligand (TRAIL) [154], and CD31 [155], or release soluble factors such IL-10, TGF-β and PGE_2_, which can inhibit effector lymphocyte function and/or DC maturation and function. It is quite possible that some of these tumor endothelial immunomodulatory mechanisms may not be directly mediated by VEGF. For example, we previoulsy found that many of the specific markers of tumor endothelium in ovarian cancer are induced not by VEGF but rather by a combination of hypoxia and inflammatory mediators [156]. Importantly, low dose cyclophosphamide used in this study to target Treg cells also exerts an antiangiogenic effect through direct cytotoxicity to tumor endothelium, which could synergize with bevacizumab to abate these aspects of the tumor endothelial barrier.

VEGF plays an important role in suppression of DC maturation. It has been demonstrated that DCs from cancer patients are functionally impaired; furthermore, the increase of immature dendritic cells in the periphery was closely correlated with serum VEGF levels but not with TGF-β, IL-6 or GM-CSF [98]. Gabrilovich and colleagues were the first to identify that VEGF released by tumor cells were capable of impairing both DC function and DC maturation from CD34+ precursors [91]. By using neutralizing blocking antibodies against VEGF (but not IL-10 or TGF-β) they were able to reverse the suppression. VEGF can exert its immune suppression on dendritic cell mostly through disruption of normal hematopoesis [90,94,95] (impairment of normal nuclear factor-kappa-B signaling during hematopoesis [92] through VEGFR-1 [56]).

Treg cells have been shown to be present in ovarian cancer [24,157]. Curiel et al. demonstrated that CD4^+^ CD25^+^ FOXP3^+^ human Treg cells suppress tumor-specific T-cell immunity and contribute to growth of human tumors in vivo in a study of 104 individuals affected with ovarian carcinoma. They found that there was a significant correlation between tumor Treg cell content and survival in the group as a, and also for individuals in stage II, , III or IV disease [158]. Presently, there are no specific drugs to deplete Treg cells, but among commercially available strategies, low-dose cyclophosphamide appears as a promising approach. In a preclinical rat model of colon cancer, Ghiringhelli et al. have shown that single administration of cyclophosphamide depletes CD4^+^ CD25^+^ T-cells, delays tumor growth, and improves cure rates when followed by non-curative immunotherapy [145]. The same group has demonstrated that metronomic oral cyclophosphamide immunosuppressive regulatory T-cells and improves effector immune function in patients with cancer [159]. Jaffee et al. showed that intravenous cyclophosphamide in doses no greater than 200 mg/m^2^ can transiently decrease Treg frequencies and enhance tumor-specific immune response in breast cancer patients in combination with a cancer vaccine [125,160].

**Figure 4:**
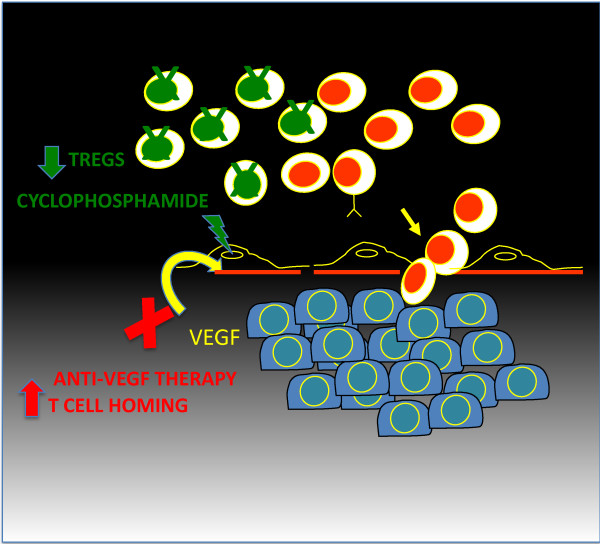
Combinatorial effect of VEGF blockade and Treg depletion.

We recently reported on a pilot study (UPCC-11807), where we administered oral metronomic cyclophosphamide at 50 mg daily every other week to patients with EOC in combination with bevacizumab. We saw no significant effect of this dose of cyclophosphamide on peripheral blood Treg. Thus, in the present study we chose the dose of 200 mg/m^2^ administered intravenously, which conveniently matches the schedule of bevacizumab and vaccine. Based on reported effects, this schedule and dose of cyclophosphamide should reduce Treg cells. The concomitant administration of intravenous low-dose cyclophosphamide and bevacizumab has never been tested before. Importantly, Treg cells and VEGF are interconnected; hypoxia (which drives expression of VEGF) induces also accumulation of CCR10^+^ Treg cells in ovarian tumors, while Treg cells can in turn reprogram the tumor microenvironment towards angiogenesis [132]. Thus, in theory although VEGF blockade as monotherapy could attenuate the blood-tumor barrier, it could also produce a rebound increase in Treg accumulation in the tumor microenvironment, promoting tolerance and angiogenesis. In this case, concomitant suppression of Treg could deprive tumors from a critical homeostatic tolerance mechanism and could produce a synergistic immunomodulatory interaction at the tumor microenvironment, allowing a relatively weak antitumor immune response induced by cancer vaccine to become clinically effective. The growing understanding of these complex networks has revealed that the same cell populations or soluble factors can simultaneously promote angiogenesis and mediate immunosuppression in the tumor microenvironment, suggesting that successful cancer vaccine therapy may indeed benefit from effective blockade of multiple mechanisms [161]. We propose to block VEGF while also suppressing Treg using readily available FDA approved drugs, such as bevacizumab and low-dose cyclophosphamide (Figure 4).

In summary, this study responds to the urgent need created by the above observations to test in the clinic a combinatorial regimen that administers cancer vaccine in combination with Treg and VEGF blockade. The present trial will enable us to take the first step in this clinical development endeavor, testing the feasibility and safety of such an approach while we collect pilot biological data from the periphery and the tumor microenvironment. Upon completion of this phase I study, we will be in a position to dissect the individual contribution of bevacizumab or cyclophosphamide to vaccine therapy through rationally designed and adequately powered phase II randomized studies, based on the results of the present study.

## Abbreviations

APC: Antigen-presenting cell; CTL: Cytotoxic T lymphocyte; CVPF: Cell and vaccine production facility; DC: Dendritic cell; DLT: Dose-limiting toxicity; DTH: Delayed type hypersensitivity; ECOG: Eastern Cooperative Oncology Group; EOC: Epithelial ovarian cancer; EOS: End of study; GM-CSF: Granulocyte/macrophage colony-stimulating factor; HOCL: Hypochlorous acid; KLH: Keyhole Limpet hemocyanin; LFTU: Long-term follow-up; OCDC: Oxidized tumor cell pulsed dendritic cell (vaccine); PBMC: Peripheral blood mononuclear cell; SAE: Serious adverse event; TAA: Tumor-associated antigen; TIL: Tumor-infiltrating lymphocytes.

## Competing interests

Authors declare that they do not have competing interest to disclose.

## Authors’ contributions

LK contributed to the conception of the study, the clinical trial design, overseeing the clinical trial and writing the manuscript, CC contributed to the preclinical data. JT is the PI of the study, GM contributed to the preclinical data, KB contributed to writing the manuscript, RM contributed to the clinical trial design and the reviewing of the manuscript. GC contributed to the conception of the study, the clinical trial design, overseeing the clinical trial and reviewing the manuscript. All authors read and approved the final manuscript.
